# A randomized controlled trial of **H**uman **P**apilloma**v**irus (HPV) testing **fo**r **c**ervic**al **cancer screening: trial design and preliminary results (HPV FOCAL Trial)

**DOI:** 10.1186/1471-2407-10-111

**Published:** 2010-03-24

**Authors:** Gina S Ogilvie, Dirk J van Niekerk, Mel Krajden, Ruth E Martin, Thomas G Ehlen, Kathy Ceballos, Stuart J Peacock, Laurie W Smith, Lisa Kan, Darrel A Cook, Wendy Mei, Gavin CE Stuart, Eduardo L Franco, Andrew J Coldman

**Affiliations:** 1 Department of Family Practice, University of British Columbia, Vancouver, Canada; 2 Division of STI/HIV Prevention and Control, BC Centre for Disease Control, Vancouver, Canada; 3 Department of Pathology and Laboratory Medicine, BC Cancer Agency, Vancouver, Canada; 4 Department of Pathology and Laboratory Medicine, University of British Columbia, Vancouver, Canada; 5 Department of Obstetrics and Gynecology, University of British Columbia, Vancouver, Canada; 6 Canadian Centre for Applied Research in Cancer Control, BC Cancer Research Centre, Vancouver Canada; 7 Population Oncology, BC Cancer Agency, Vancouver, Canada; 8 Cervical Cancer Screening Program, BC Cancer Agency, Vancouver, Canada; 9 Hepatitis Services, BC Centre for Disease Control, Vancouver, Canada; 10 Clinical Trials Unit, Laboratory Services, Provincial Health Services Authority; 11 Division of Cancer Epidemiology, McGill University, Montreal, Canada

## Abstract

**Background:**

In the HPV FOCAL trial, we will establish the efficacy of hr-HPV DNA testing as a stand-alone screening test followed by liquid based cytology (LBC) triage of hr-HPV-positive women compared to LBC followed by hr-HPV triage with ≥ CIN3 as the outcome.

**Methods/Design:**

HPV-FOCAL is a randomized, controlled, three-armed study over a four year period conducted in British Columbia. It will recruit 33,000 women aged 25-65 through the province's population based cervical cancer screening program. *Control arm: *LBC at entry and two years, and combined LBC and hr-HPV at four years among those with initial negative results and hr-HPV triage of ASCUS cases; *Two Year Safety Check arm*: hr-HPV at entry and LBC at two years in those with initial negative results with LBC triage of hr-HPV positives; *Four Year Intervention Arm*: hr-HPV at entry and combined hr-HPV and LBC at four years among those with initial negative results with LBC triage of hr-HPV positive cases

**Discussion:**

To date, 6150 participants have a completed sample and epidemiologic questionnaire. Of the 2019 women enrolled in the control arm, 1908 (94.5%) were cytology negative. Women aged 25-29 had the highest rates of HSIL (1.4%). In the safety arm 92.2% of women were hr-HPV negative, with the highest rate of hr-HPV positivity found in 25-29 year old women (23.5%). Similar results were obtained in the intervention arm HPV FOCAL is the first randomized trial in North America to examine hr-HPV testing as the primary screen for cervical cancer within a population-based cervical cancer screening program.

**Trial Registration:**

International Standard Randomised Controlled Trial Number Register, ISRCTN79347302

## Background

Cervical cancer screening using cervical cytology (the Pap smear) has been an extremely successful public health intervention, achieving reductions in cervical cancer incidence of up to 80% where practiced effectively[1]. However, the Pap smear was introduced over 50 years ago and studies have now proven it has significant limitations. Data from some jurisdictions indicate that cervical cancer rates have reached a nadir, and meta-analyses indicate that the sensitivity of a single Pap test to detect cervical intraepithelial neoplasia (CIN) or invasive cervical cancer is less than 60%[2,3].

There is now ample evidence that infection with high-risk types of the Human Papillomavirus (hr-HPV) is a requisite intermediate step for the development of cervical cancer and its precursors[4,5]. On this basis, it has been proposed that testing for the presence of hr-HPV could improve cervical cancer screening. HPV testing is recommended for follow up of abnormal cytology in women over the age of 30 and for the surveillance of patients after colposcopic treatment for CIN[6]. When used as a primary screening tool in cross sectional studies, it has been demonstrated that hr-HPV testing has a higher sensitivity and negative predictive value (NPV) for CIN2 or worse (≥ CIN2) detection than either the conventional Pap smear or liquid based cytology (LBC), albeit with a lower specificity and positive predictive value (PPV)[7-12]. In recognition of this, one approach for screening would be to use hr-HPV testing as a **single primary screening test **with cytology reserved only for the triage of women having a positive test, especially following the advent of HPV vaccination[12,13]. This would offer several advantages over combined testing:

• Screening would be undertaken with the test having higher *sensitivity *(hr-HPV testing);

• 85-90% of women would be returned immediately to routine screening with a negative hr-HPV test without incurring the cost of cytology, which would be reserved only for those with a positive hr-HPV;

• The high-volume screening of samples would be undertaken with a non-subjective test that can be automated, while the subjective, labour-intensive test would be restricted to high-risk samples that could be examined with greater vigilance because of the reduced number to be interpreted;

• It represents a more robust screening approach that could serve the additional purpose of post-vaccination surveillance in the population[13];

• The recommended cervical cancer screening interval can be extended, as the long term risk of CIN3 or worse in women with a negative hr-HPV test is much lower than those who have a negative cytology, thus providing greater reassurance to women and also resulting in potential cost savings[10].

To examine these concepts, several international large randomized controlled trials (RCT) are being conducted in Europe and in Canada to evaluate HPV testing as part of primary cervical cancer screening[9,14-21]. With the exception of the Finnish Randomized Public Health Trial and phase 2 of the New Technologies for Cervical Cancer Screening (NTCC) trial in Italy, these trials have compared combined HPV and cytology testing vs. cytology alone as the primary screening intervention. The Phase 2 of NTCC and the Finnish trials are comparing HPV versus cytology as the primary screen, and both of these employ conventional cytology as opposed to liquid based cytology (LBC) which is replacing conventional cytology in several jurisdictions[14,20]. To date, there has not been a RCT of hr-HPV detection followed by cytology triage of hr-HPV positive women, compared to cytology alone in a population based screening program in North America, and no trials have utilized LBC. It is essential to properly evaluate this approach within the context of a population based cervical cancer screening program which would provide generalizable evidence to inform policy decisions concerning cervical cancer screening internationally.

This paper describes the design and preliminary screening results of the HPV FOCAL Trial. The primary objective of the HPV FOCAL trial is to establish the efficacy of hr-HPV testing followed by liquid based cytology (LBC) triage of hr-HPV-positive women compared to LBC followed by hr-HPV triage for cervical cancer screening with ≥ CIN3 as the outcome, through a comparison of the estimated decreases in cervical intraepithelial neoplasia (CIN 2/3) that can be achieved by each screening modality in successive screening rounds. The secondary objectives of this trial are to establish the appropriate screening interval for hr-HPV negative women, using the current standard of a 2-year recall interval for cytology negative women as the benchmark of acceptable risk in British Columbia; to establish the appropriate clinical follow-up for hr-HPV positive women; and to establish the cost-effectiveness of hr-HPV testing for primary screening, all within the context of a population based Canadian cervical cancer screening program. The results of this trial will demonstrate whether or not the use of hr-HPV testing as a single primary screening test within a population based cervical cancer screening program will be able to provide further reductions in the incidence of cervical cancer and its precursor lesions, allow the screening interval to be extended, and improve the cost-effectiveness of cervical cancer screening.

## Methods/Design

### Trial Design

**Figure 1:**
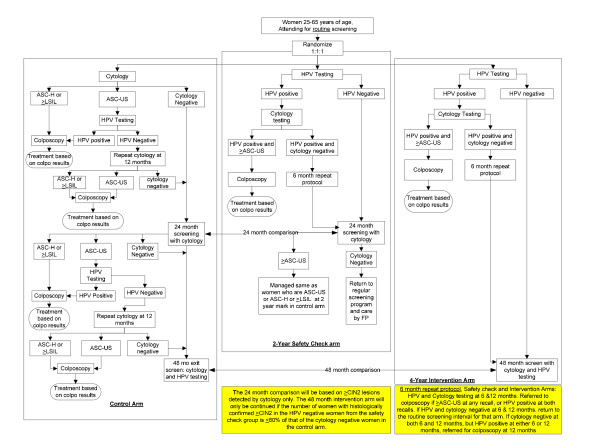
**HPV FOCAL: a three-armed, RCT over a four year period**.

HPV FOCAL is a three-armed, RCT over a four year period (Figure 1).

**Control arm**: LBC at entry and again at two years; ASCUS cases are triaged with hr-HPV testing; combined LBC and hr-HPV at four years (exit screen) among those with initial negative results.

**Two Year Safety Check arm**: hr-HPV at entry; LBC at two years (exit screen) in those with initial negative hr-HPV results with LBC triage of hr-HPV positives at either round.

**Four Year Intervention Arm**: hr-HPV at entry with LBC triage of hr-HPV positives; combined hr-HPV and LBC at four years (exit screen) among those with initial hr-HPV negative results.

### British Columbia Population Based Cervical Cancer Screening Program

Since the 1960s, cervical cancer screening in British Columbia has been organized as a centrally administered program[22]. A single provincial laboratory (Provincial Health Services Laboratories) processes and interprets Pap test samples from all clinicians in the province. A unified set of recommendations for the management of women with abnormal Pap tests is produced by the British Columbia Cancer Agency (BCCA), and is communicated to the healthcare providers with the Pap test results. BCCA also manages a coordinated follow-up system involving regional colposcopy clinics across the province for women with abnormal Pap tests. BCCA's Cervical Cancer Screening Program maintains a single data structure linking all Pap test results and disease outcomes, and provides overall program administration and coordination of promotion, recruitment, follow-up reminder system, follow-up tracking, quality management, program evaluation and research support.

### Study Population and Recruitment

Women aged 25 to 65, registered with Medical Services Plan in British Columbia, who receive care from a participating family physician (FP) for routine cervical screening are eligible. Exclusion criteria are: a history of histologically proven CIN2 or worse requiring treatment in the last five years; a history of histologically proven invasive cervical cancer; a Pap smear within the preceding twelve months; no cervix; pregnant at time of enrolment; HIV positive or on immunosuppressive treatments; or unwilling or unable to provide informed consent.

Women are invited to participate in the study when they present for cervical cancer screening and are deemed eligible to participate by their FP or when pre-identified as being due for screening from the BCCA centralized provincial cytology database. For the pre-identified, the FP office sends the woman a study package that includes an invitation letter, a study information pamphlet and the informed consent form. The invitation letter requests women to phone their FP to make an appointment for their cervical screening test and also provides them with the opportunity to contact, or be contacted by study staff to learn more about the trial and decide on participation. All participants are consented by their FP and are asked to complete an epidemiologic questionnaire. A financial implications questionnaire is also completed by a random sample of participants. Full cervical cancer screening history is available through data linkage to the cervical cancer screening program, and HPV vaccination status is self reported.

### Study Protocol (Figure 1)

In addition to the control arm and the intervention arm, a 'two year safety-check' arm is included in this trial. At the time of the trial design, there were ethical concerns identified in changing the screening interval at the same time as changing the primary screening tool in a clinical trial. To that end, we included a safety check arm, to examine the safety of hr-HPV testing within the usual screening interval. This information will be used by the data safety monitoring board to verify the safety of hr-HPV testing at the two year interval and infer the suitability of the intervention arm as the trial proceeds.

Two samples are collected during the initial screening appointment. Specimen1 (LBC) is collected first with the ThinPrep^® ^Broom-like collection device and is placed in a ThinPrep^® ^PreservCyt vial (Hologic Inc, Bedford MA) and is used for all the HPV FOCAL trial testing. Specimen 2 is collected in Digene STM^® ^(Qiagen, Mississauga ON) and is frozen for future use. Both specimens are sent to the central laboratory in Vancouver, for trial testing (specimen 1) and for storage (specimen 2). Upon sample receipt, the woman is randomized to one of the three study arms and specimen 1 is aliquoted (Figure 1). For those allocated to hr-HPV testing arms, an aliquot is removed and processed using the Qiagen sample conversion kit, and tested using the Digene Hybrid Capture 2 (HC2) assay (Qiagen, Mississauga ON), which tests simultaneously for the presence of DNA from 13 hr-HPV types. Results are classified as hr-HPV negative, hr-HPV positive or unsatisfactory. LBC testing is conducted on specimen 1 using the ThinPrep^® ^collected device, according to the manufacturer's recommendations. Cytological evaluation and reporting follow the Bethesda classification system [23].

#### Colposcopy

If the woman's test results indicate that a colposcopy referral is recommended (Figure 1), this will be conveyed to the FP with the study sample results. All colposcopy examinations are performed at study-designated colposcopy clinics in Vancouver and Victoria to ensure consistency in diagnostic performance. Colposcopic examinations performed in British Columbia are highly standardized, and clinicians providing colposcopy adhere to a study-agreed protocol.

Women referred for colposcopy fall into the following major groupings:

a) Cytology: AGC (hr-HPV positive or not done);

b) Cytology: ASC-H or ≥ LSIL (hr-HPV positive or not done);

c) Cytology: ASC-US; (hr-HPV positive or persistent ASC-US which was initially hr-HPV negative).

Women are managed according to the standard provincial guidelines in the province of British Columbia[24]. Colposcopy is used to assess the highest grade lesion seen on the cervix and directed biopsy(s) is performed as well as an endocervical curettage when appropriate. The standard treatment for CIN2+ in British Columbia is an excisional treatment, most commonly loop electrosurgical excision procedure (LEEP) and occasionally cone biopsy

#### Histology

Pathological interpretation of biopsies is conducted at two centres which provide services to the participating colposcopy clinics. Pathologists are blinded to cytology and hr-HPV result when interpreting the slides. Histology results are stored in the same Laboratory Information System (LIS) as the cytology results.

Following standard practice, if there is significant discordance between the cytologic and histologic evaluation which would potentially influence patient management, the colposcopist contacts the laboratory to request review and correlation of the histology and cytology in order to resolve the discrepancy (e.g. a case where HSIL cytology is noted, but negative histology,) and determine an appropriate disease management strategy.

A review of exit screen histology is an essential component of the trial. Approximately 50% of exit screen histology results will undergo second review by senior study pathologists. If the primary and review histology results agree, this will be the final study diagnosis, but in the case of disagreement between primary and review histology, the slide will be referred to another senior pathologist. If this result agrees with either the primary result or first review the agreed results will be the final study diagnosis. If all 3 results are different, the final study diagnosis will be established by consensus between all three pathologists.

### Randomization and Blinding

A database has been developed specifically for the HPV FOCAL trial. Randomization occurs through this database when samples are received at the laboratory. At the time of randomization a Study Identification Number (SIN) is allocated to the participant. Samples are stratified by age and simple equal (1/3 probability) random allocation occurs at the laboratory. Upon randomization, study staff label and batch the specimens for initial processing (hr-HPV or LBC).

FPs and participants are blinded to study arm allocation. Participant results are communicated from the BCCA to the FP office as soon as they are known. If the participant's initial screening results (hr-HPV or cytology) are "negative" the report states "within normal limits". The report also states that the recommended follow-up for that participant will be communicated to the FP in two years' time. This ensures blinding is maintained for as long as possible and prevents any bias that may potentially occur from knowledge of negative results. If the screening results are hr-HPV and/or cytology positive, the results are communicated from the BCCA to the FP along with the recommended follow-up.

### Statistical Considerations

#### Sample Size

The planned intake sample size is 11,000 women per arm (33,000 total). Power calculations were performed using the nQuery Advisor, version 2, software package. Rates of hr-HPV infection and associated histologically proven disease were based on results from the HART study[9], prevalence rates from the CCCaST trial conducted in Quebec and Newfoundland[8,25]. Sample sizes were based on requiring at least an 80% power to detect relative differences (alternative hypothesis) of 20% in the outcome comparisons for intervention versus control at four years and control versus safety check arms at two years.

### Analysis

#### Primary Outcome Measures

• Control and intervention arms: Cumulative incident ≥ CIN3 detected up to and including four years in both the control arm and the intervention arm

• Control and safety check arms: Incident ≥ CIN2 detected at two years. If the number of ≥ CIN2 in the safety-check group exceeds 0.8 times that in the control arm at the 2 year screen, then the trial will conclude and women in the four year intervention arm will be recalled at two years for their exit screen.

#### Secondary Outcome Measures

• Rates of ≥ CIN2 and hr-HPV respectively at initial screen in control and safety/intervention arms;

• Rates of incident ≥ CIN2 at two years in control arm and at four years in intervention arm;

• The total estimated cost per woman screened and the total estimated cost per quality-adjusted life-year gained for each technology;

• Clearance of hr-HPV infection in women who are hr-HPV-positive and cytology negative at initial screen.

The primary outcome analysis will be a comparison of histologically confirmed ≥ CIN3 between the intervention and control arms. Rates of lesion occurrence will be calculated using person-time denominators for the different study intervals and compared via Kaplan-Meier plots[26]. Rates will be calculated for specific age groups within the study. Significance testing will be based on Poisson statistics and performed at the 5% level (2-sided). Analysis will also be performed using logistic regression to permit control of potential confounding factors since for some comparisons balance may not be assured by randomization. The primary comparison of disease rates between test negative groups (at entry screen) will not control for potential confounding factors since they are not balanced by randomization and the tests may select for different characteristics. Covariate-adjusted analyses will be performed to determine the extent to which any difference is explicable by potential confounders (e.g., age and sexual behaviour).

### Ethical Issues

This study is being conducted in accordance with the Ethical Conduct for Research Involving Humans Tri-Council Policy Statement http://www.nserc-crsng.gc.ca/NSERC-CRSNG/governance-gouvernance/ethics-ethiques_eng.asp. Ethics approval has been obtained from appropriate local research ethics boards. The trial registration number is International Standard Randomised Controlled Trial Number Register: ISRCTN79347302. All information about this trial is kept behind locked doors or in secure computer files. On interim and final reports, all data will be de-identified.

## Discussion

### Preliminary Findings

Recruitment for trial participants commenced in December 21, 2007 through 147 FP offices. As of December 31, 2009, 37,347 women were identified as potentially eligible through the Cervical Cancer Screening Program database. Of the above identified, 28,525 women were sent invitation letters from their family physicians to participate in this study, 613 were ineligible and 2995 declined participation (data on non responders not available). 9842 women were enrolled in the trial through 147 FP clinics from Vancouver Island and Metro Vancouver and 6150 had completed both the epidemiology questionnaire (Epi-Q) and had a preliminary study specimen result. Data will be presented on women who had both a study specimen result and completed Epi-Q.

Participant age ranged from 25 to 65 years, with a median age of 45 (Table 1). As part of trial eligibility, women do not have a history of invasive cancer and have not had > or = CIN2 requiring treatment within the last 5 years. The majority of women commenced sexual intercourse prior to the age of 19 and over 90% reported their sexual debut by the age of 24. Just over sixty percent of women had never smoked (Table 2). The distribution of study characteristics among trial arms was well balanced.

**Table 1:** Socio-demographic Characteristics of 6150 HPV FOCAL Participants by Study Arm

	Arm			
	**Control (%)**	**Safety (%)**	**Intervention (%)**	**Total (%)**

**Age Group (years)**				

25-29	141 (7%)	153 (7.2%)	127 (6.3%)	421 (6.8%)

30-34	172 (8.5%)	172 (8.1%)	166 (8.3%)	510 (8.3%)

35-39	284 (14.1%)	315 (14.9%)	269 (13.4%)	868 (14.1%)

40-44	318 (15.8%)	351 (16.6%)	319 (15.9%)	988 (16.1%)

45-49	357 (17.7%)	371 (17.5%)	376 (18.7%)	1104 (18%)

50-54	309 (15.3%)	312 (14.7%)	283 (14.1%)	904 (14.7%)

55-59	252 (12.5%)	241 (11.4%)	279 (13.9%)	772 (12.6%)

60-65	186 (9.2%)	204 (9.6%)	193 (9.6%)	583 (9.5%)

**Cultural Group**				

Aboriginal	61 (3%)	58 (2.7%)	48 (2.4%)	167 (2.7%)

Black	10 (0.5%)	10 (0.5%)	7 (0.3%)	27 (0.4%)

British	1113 (55.1%)	1178 (55.6%)	1118 (55.6%)	3409 (55.4%)

Chinese	181 (9%)	211 (10%)	198 (9.8%)	590 (9.6%)

French	196 (9.7%)	178 (8.4%)	174 (8.6%)	548 (8.9%)

Southeast Asian	9 (0.4%)	8 (0.4%)	4 (0.2%)	21 (0.3%)

Northern European	136 (6.7%)	144 (6.8%)	160 (8%)	440 (7.2%)

Southern European	135 (6.7%)	119 (5.6%)	97 (4.8%)	351 (5.7%)

Eastern European	251 (12.4%)	259 (12.2%)	243 (12.1%)	753 (12.2%)

Western European	316 (15.7%)	355 (16.8%)	335 (16.7%)	1006 (16.4%)

Other	316 (15.7%)	320 (15.1%)	282 (14%)	918 (14.9%)

**Marital Status**				

Divorced	176 (8.8%)	209 (9.9%)	212 (10.6%)	597 (9.8%)

Married	1324 (66.3%)	1381 (65.7%)	1274 (64%)	3979 (65.3%)

Single	286 (14.3%)	292 (13.9%)	285 (14.3%)	863 (14.2%)

Widowed	30 (1.5%)	25 (1.2%)	25 (1.3%)	80 (1.3%)

Common-law	182 (9.1%)	195 (9.3%)	196 (9.8%)	573 (9.4%)

**Educational History**				

Elementary/Incomplete High School	41 (2.1%)	55 (2.6%)	46 (2.3%)	142 (2.3%)

High School (complete)	296 (14.8%)	292 (14%)	283 (14.3%)	871 (14.3%)

Trade Certificate/College	591 (29.6%)	629 (30.1%)	608 (30.6%)	1828 (30.1%)

University (incomplete)	134 (6.7%)	121 (5.8%)	131 (6.6%)	386 (6.4%)

University graduate or higher	935 (46.8%)	991 (47.5%)	917 (46.2%)	2843 (46.8%)

**Employment**				

Currently Working	1588 (78.8%)	1666 (78.8%)	1556 (77.7%)	4810 (78.5%)

On Disability	58 (2.9%)	61 (2.9%)	49 (2.5%)	168 (2.8%)

On Social Assistance	4 (0.2%)	7 (0.3%)	9 (0.5%)	20 (0.3%)

**Table 2:** Life style, Pregnancy and Sexual Characteristics of 6150 HPV FOCAL Participants by Study Arm

	Arm			
	**Control (%)**	**Safety (%)**	**Intervention (%)**	**Total (%)**

**Smoked Regularly (Ever)**	790 (41.3%)	746 (37.7%)	764 (40.4%)	2300 (39.8%)

**Mean Age of Started Smoking in Years (N = 2300)**	16.4	16.6	16.5	16.5

Current Smoker	150 (19.1%)	135 (18.2%)	144 (18.8%)	429 (18.7%)

**History of Sexual Intercourse**				

Never sexually active	8 (0.4%)	9 (0.4%)	6 (0.3%)	23 (0.4%)

**Mean Age of Sexual Debut in Years (N = 5984)**	18.8	18.8	18.7	18.8

**Lifetime No. Male Sexual Partners (N = 6013)**				

0	1 (0.1%)	4 (0.2%)	4 (0.2%)	9 (0.1%)

1	420 (21.2%)	429 (20.8%)	422 (21.4%)	1271 (21.1%)

2-5	650 (32.9%)	757 (36.6%)	685 (34.8%)	2092 (34.8%)

6-10	482 (24.4%)	458 (22.2%)	458 (23.3%)	1398 (23.2%)

11-50	391 (19.8%)	393 (19%)	376 (19.1%)	1160 (19.3%)

51-99	22 (1.1%)	23 (1.1%)	22 (1.1%)	67 (1.1%)

99+	11 (0.6%)	3 (0.1%)	2 (0.1%)	16 (0.3%)

**Male Sexual Partners in Past Six Months (N = 5990)**				

0	294 (14.9%)	322 (15.6%)	324 (16.6%)	940 (15.7%)

1	1618 (82.1%)	1681 (81.4%)	1586 (81.2%)	4885 (81.6%)

2+	59 (3%)	62 (3%)	44 (2.3%)	165 (2.8%)

**History of Pregnancy**				

Ever Pregnant before (N = 6004)	1588 (81.1%)	1654 (79.9%)	1602 (81.1%)	4844 (80.7%)

**Mean age at first pregnancy (N = 4844)**	26.5	26.5	26.3	26.5

**Mean Number of Pregnancies (N = 4844)**	2.7	2.6	2.7	2.7

**Mean Age of Menarche (N = 6087)**	12.8	12.9	12.8	12.8

**Oral Contraceptive**				

Ever	1765 (87.4%)	1824 (86.1%)	1743 (86.6%)	5332 (86.7%)

**Mean Years on Oral Contraceptives (N = 5332)**	9.0	8.9	9.0	9.0

**Contraception Method**				

Barrier (Ever)	1550 (76.8%)	1638 (77.3%)	1529 (76%)	4717 (76.7%)

Barrier (Current)	276 (13.7%)	293 (13.8%)	253 (12.6%)	822 (13.4%)

Vaginal (Ever)	197 (9.8%)	269 (12.7%)	243 (12.1%)	709 (11.5%)

Vaginal (Current)	7 (0.3%)	10 (0.5%)	11 (0.5%)	28 (0.5%)

Hormonal (Ever)	1771 (87.7%)	1829 (86.3%)	1746 (86.8%)	5346 (86.9%)

Hormonal (Current)	286 (14.2%)	291 (13.7%)	268 (13.3%)	845 (13.7%)

Permanent (Ever)	659 (32.6%)	689 (32.5%)	654 (32.5%)	2002 (32.6%)

Permanent (Current)	576 (28.5%)	626 (29.5%)	556 (27.6%)	1758 (28.6%)

Rhythm/Withdrawal (Ever)	204 (10.1%)	222 (10.5%)	176 (8.7%)	602 (9.8%)

Rhythm/withdrawal (Current)	53 (2.6%)	54 (2.5%)	39 (1.9%)	146 (2.4%)

**Duration of HRT for Menopause in Years (N = 241)**	4.2	4.5	5.6	4.7

Of the 2019 women enrolled in the control arm, 1908 (94.5%) were cytology negative. Sixteen women (0.8%) had high grade squamous intraepithelial lesions (HSIL) on their cytology evaluations, and women aged 25-29 had the highest rates of HSIL (1.4%) (Table 3). In the safety arm 92.2% of women were hr-HPV negative. The highest rate of hr-HPV positivity was in 25-29 year old women (23.5%) and the lowest rate was found in women aged 55-59 (3.7%). Similar results were obtained in the intervention arm, where 92.1% were hr-HPV negative and the highest rates of hr-HPV positivity (24.4%) were found in women aged 25-29 (Table 4).

**Table 3:** Results of screening by Five Year Age Strata: Control (cytology) arm

Age Strata	Cytology Negative (%)	Cytology ASC-US (%)	Cytology ATY (%)	Cytology LSIL (mild) (%)	Cytology ASC-H (%)	Cytology HSIL (%)	Cytology Smear Unsatisfactory (%)	Total
25-29	123 (87.2)	4 (2.8)	0 (0.0)	6 (4.3)	2 (1.4)	2 (1.4)	4 (2.8)	141

30-34	157 (91.3)	1 (0.6)	1 (0.6)	9 (5.2)	0 (0.0)	2 (1.2)	2 (1.2)	172

35-39	270 (95.1)	6 (2.1)	0 (0.0)	5 (1.8)	1 (0.4)	1 (0.4)	1 (0.4)	284

40-44	302 (95.0)	3 (0.9)	1 (0.3)	6 (1.9)	0 (0.0)	3 (0.9)	3 (0.9)	318

45-49	336 (94.1)	9 (2.5)	0 (0.0)	5 (1.4)	1 (0.3)	4 (1.1)	2 (0.6)	357

50-54	296 (95.8)	4 (1.3)	0 (0.0)	2 (0.6)	2 (0.6)	2 (0.6)	2 (0.6)	309

55-59	244 (96.8)	0 (0.0)	0 (0.0)	3 (1.2)	0 (0.0)	0 (0.0)	5 (2.0)	252

60+	180 (96.8)	1 (0.5)	1 (0.5)	0 (0.0)	1 (0.5)	2 (1.1)	1 (0.5)	186

Total	1908 (94.5)	28 (1.4)	4 (0.2)	36 (1.8)	7 (0.3)	16 (0.8)	20 (1.0)	2019

ASC-US - Atypical squamous cells of undetermined significance.

ATY - Atypical Glandular Cells/NOS; Atypical Endometrial Cells/NOS; Atypical Endocervical Cells/NOS

LSIL - Low-grade squamous intraepithelial lesion

ASC-H - Atypical squamous cells cannot exclude high-grade squamous intraepithelial lesion.

HSIL - High-grade squamous intraepithelial lesion (moderate, severe dysplasia, Ca in situ)

**Table 4:** Results of screening by Five Year Age Strata: Safety Check and Intervention arms

	Safety Check Arm				Intervention Arm			
**Age Strata**	**hr-HPV Negative (%)**	**hr-HPV Positive (%)**	**hr-HPV Unsatis (%)**	**Total**	**hr-HPV Negative (%)**	**hr-HPV Positive (%)**	**hr-HPV Unsatis (%)**	**Total**

25-29	117 (76.5)	36 (23.5)	0 (0.0)	153	95 (74.8)	31 (24.4)	1 (0.8)	127

30-34	151 (87.8)	21 (12.2)	0 (0.0)	172	146 (88.0)	20 (12.0)	0 (0.0)	166

35-39	284 (90.2)	31 (9.8)	0 (0.0)	315	239 (88.8)	30 (11.2)	0 (0.0)	269

40-44	324 (92.3)	26 (7.4)	1 (0.3)	351	300 (94.0)	18 (5.6)	1 (0.3)	319

45-49	358 (96.5)	13 (3.5)	0 (0.0)	371	354 (94.1)	19 (5.1)	3 (0.8)	376

50-54	293 (93.9)	18 (5.8)	1 (0.3)	312	267 (94.3)	15 (5.3)	1 (0.4)	283

55-59	232 (96.3)	9 (3.7)	0 (0.0)	241	265 (95.0)	13 (4.7)	1 (0.4)	279

60+	195 (95.6)	9 (4.4)	0 (0.0)	204	188 (97.4)	4 (2.1)	1 (0.5)	193

Total	1954 (92.2)	163 (7.7)	2 (0.1)	2119	1854 (92.1)	150 (7.5)	8 (0.4)	2012

HPV Digene Negative: Non-reactive for HPV High or Intermediate risk types.

HPV Digene Positive: Reactive for HPV High or Intermediate risk types.

HPV Digene Unsatis: Results unsatisfactory - need to repeat specimen

The HPV FOCAL study is the first RCT to examine hr-HPV testing followed by LBC triage compared to LBC followed by hr-HPV triage as the primary screen for cervical cancer within the context of a population based cervical cancer screening program in North America. This study will provide generalizable evidence to inform policy decisions concerning cervical cancer screening internationally, particularly in settings where LBC has already been adopted or is under consideration. Participants are women who are part of a long established cervical cancer screening program and represent a population based cohort of women at average risk for cervical cancer in North America. Study characteristics for the first 6,000 participants were well balanced across the three trial arms. In each arm of the trial, over 90% of women were negative on their screening examination, with the majority of abnormalities found in women in under the age of 35.

Important design elements in HPV FOCAL include the use of LBC (a more universal cytology format in North America) as opposed to conventional cytology; the use of FPs as study collaborators to recruit participants; blinded analysis of cytology and pathology results with respect to hr-HPV status; use of a single laboratory for cytology analysis; inclusion of a two year safety arm and standardized colposcopy protocols. The use of LBC is of particular relevance because of the ability to use the sample for reflex testing. LBC, which involves taking a sample and placing it into a vial with liquid, and then producing a slide for examination from a suspension of the cells has been compared to conventional cytology in many studies[27]. Studies have reported higher sensitivity with LBC compared to conventional smears, as well as a greater proportion of adequate specimens for evaluation. However, there remains a divergence of opinion on the advantages of LBC over conventional cytology, and a recent RCT found that LBC had equivalent sensitivity for ≥ CIN2 lesions relative to conventional Pap, but had a lower rate of unsatisfactory smears when compared with the latter[28]. In a cluster randomized trial involving over 89,000 women, Siebers et al. [29] found that LBC did not perform any better than well-performed conventional Pap smears for the detection of cervical cancer precursors. Despite the differences in results, LBC has been widely adopted worldwide and particularly in North America as a part of cervical cancer screening programs due to opportunities for automation, improved costs and single specimen collection for HPV and other molecular tests. The HPV FOCAL trial is well positioned to examine the contribution of LBC with respect to primary HPV testing and inform the discussion internationally. These findings would be directly relevant to cervical cancer screening programs worldwide as they consider the respective roles of LBC and HPV testing in their future program delivery.

Findings from this preliminary analysis contribute to available literature on the prevalence of HPV in Canadian women. In this study of women recruited from a population based screening program, eight percent were positive for hr-HPV, with the highest prevalence rates being found in women aged 25-29. In the first Canadian study comparing Pap screening with hr-HPV screening, women aged 30-69 from Newfoundland and Quebec presenting for routine cervical cancer screening found to have an hr-HPV prevalence of 6.1% overall[8,19,25]. In another study of hr-HPV prevalence in women in the cervical cancer screening program in British Columbia, Moore et al., using GP5+/GP6+ L1 consensus primers, found a much higher hr-HPV prevalence of 13.9%[30]. Differences between Moore's study and this study may be attributed to differing recruitment methods (population- vs. clinic-based) and different laboratory methods for establishing presence or absence of hr-HPV. Further afield, hr-HPV prevalence has ranged from 2.2% to 15.7% in several European studies of women attending routine cervical screening[31]. As with this study, the peak prevalence for hr-HPV is overall was found in women between the ages of 25 and 34.

In a recent joint analysis of several European cohort studies and clinical trials, Dillner found that hr-HPV results had a high negative predictive value (99%), with women who were negative for hr-HPV having a very low risk of development of ≥ CIN3 in the next six years[10]. This study concludes that this low six year cumulative incidence rate for ≥ CIN3 among hr-HPV negative women suggests that cervical cancer screening intervals could be safely extended. In HPV FOCAL, by using a safety check arm of two years, we will be able to examine in a controlled setting whether screening programs can safely extend screening intervals from the traditional one or two years used around the world, and confirm the recommendations of the European consortium study.

Data from ongoing randomized trials comparing primary screening for cervical cancer with HPV and cytology have found that HPV-based screening offers improved sensitivity for high grade CIN lesions, but with a commensurate loss in specificity. In Dillner's review[10], he noted that most cohort studies and randomized trials were limited by the number of ≥ CIN3 cases in the studies, thus reducing the statistical power to examine the screening interval using ≥ CIN3 as the endpoint. In this study, sample size calculations were conducted with ≥ CIN3 as the endpoint, resulting in the very large sample size (over 30,000 women) with the intention of having enough power to compare the incident rates of ≥ CIN3 in the control arm compared to the intervention arm. The expectation is that eventual findings of the HPV FOCAL trial will contribute to the growing body of literature examining the rapidly shifting paradigm of cervical cancer screening in the era of HPV-based technologies.

## Competing interests

* ELF has served as occasional consultant to biotechnology companies involved with cervical cytology (Ikonisys, Cytyc) and HPV testing (Roche, Qiagen, Gen-Probe).

## Authors' contributions

GSO, DVN, MK, REM, TGE, LK, SP, GCES, ELF and AJC all contributed to the conception, design and secured funding for the study. DVN, MK, KC, LK, DAC, WM lead and carried out cytology and molecular studies in the laboratory. GSO, MK, REM, LS, LK, ELF and AJC developed and conducted the epidemiological and statistical analysis. GSO, MK, REM, TGE, LS, WM, DAC, ELF and AJC drafted the preliminary manuscript. DVN, LK, GCES, KC, SP made substantive revisions to the preliminary version. All authors read and approved the final manuscript.

## Pre-publication history

The pre-publication history for this paper can be accessed here:

http://www.biomedcentral.com/1471-2407/10/111/prepub
